# Safety and immunogenicity of an attenuated Chinese pseudorabies variant by dual deletion of TK&gE genes

**DOI:** 10.1186/s12917-018-1536-7

**Published:** 2018-09-21

**Authors:** Jichun Wang, Zengcai Song, Aimin Ge, Rongli Guo, Yongfeng Qiao, Mengwei Xu, Zhisheng Wang, Yamei Liu, Yating Zheng, Hongjie Fan, Jibo Hou

**Affiliations:** 1 National Research Center of Engineering and Technology for Veterinary Biologicals/Institute of Veterinary Immunology & Engineering, Jiangsu Academy of Agricultural Sciences, Nanjing, 210014 Jiangsu China; 2 Jiangsu Co-innovation Center for Prevention and Control of Important Animal Infectious Diseases and Zoonoses, Yangzhou, China; 3 College of Veterinary Medicine, Nanjing Agricultural University, Nanjing, 210095 China; 4 Shandong Vocational Animal Science and Veterinary College, Weifang, 261061 China; 5 Institute of Veterinary Medicine, Jiangsu Academy of Agricultural Sciences, Nanjing, 210014 Jiangsu China

**Keywords:** Pseudorabies virus emerging variant, TK&gE dual deletion, Attenuation, Live vaccine, Safety, Immunogenicity

## Abstract

**Background:**

Since the outbreak of a new emerging virulent pseudorabies virus mutant in Chinese pig herds, intensive research has been focused on the construction of novel gene deletion vaccine based on the variant virulent viruses. An ideal vaccine candidate is expected to have a balanced safety and immunogenicity.

**Results:**

From the infectious clone of PRV AH02LA strain, a TK deletion mutant was generated through two-step Red mutagenesis. After homologous recombination with a transfer vector, a TK&gE dual deficient mutant PRV (PRV^ΔTK&gE-AH02^) was generated, and its structure verified by PCR, RFLP and sequencing. Growth kinetics test showed that PRV^ΔTK&gE-AH02^ reached a titer of 10^7.5^ TCID_50_ /mL on ST cells.

The PRV^ΔTK&gE-AH02^ at a dose of 10^6.0^ TCID_50_ /animal was not virulent in mice or 1-day-old piglets with maternal PRV antibodies. No clinical signs or virus shedding were detected in 28~ 35-day-old piglets without maternal PRV antibodies after nasal or intramuscular administration with a dose of 10^6.0^ TCID_50_, although it caused one death of four 1-day-old piglets without maternal PRV antibodies. In the efficiency test of PRV^ΔTK&gE-AH02^, all four 28~ 35-day-old piglets without PRV antibody in the challenge control showed typical clinical symptoms and virus shedding, and two died at 4~ 5 days post challenge. All piglets in 10^5.0^, 10^4.0^ and 10^3.0^ TCID_50_/dose PRV^ΔTK&gE-AH02^ groups provided complete protection against challenge at only 7 days post intramuscular vaccination. More importantly, PRV^ΔTK&gE-AH02^ stopped virus shedding in these piglets. In contrast, all four piglets in PRV Bartha K61 vaccine group developed high body temperature (≥40.5 °C) and viral shedding, despite they had mild or even no clinical symptoms.

**Conclusions:**

The constructed TK&gE dual deletion mutant PRV^ΔTK&gE-AH02^ can reach high titers on ST cells. The live vaccine of PRV^ΔTK&gE-AH02^ is highly safe, and can not only provide clinical protection but also stops virus shedding. This study suggests that PRV^ΔTK&gE-AH02^ might work as a promising vaccine candidate to combat the PRV variant emerging in Chinese herds since 2011.

## Background

Since 2011, a new emerging pseudorabies virus(PRV) variant has swept many Chinese pig herds, leading to infection or disease of variable severity [1–4]. Several studies have shown that PRV Bartha K61 vaccine can only provide clinical protection against the new PRV mutants, but fail to stop virus shedding in piglets post challenge [5]. It is therefore urgent to develop a more efficacious vaccine in order to eradicate the virulent PRV variant. Since successful eradication of pseudorabies in many nations have been achieved via the application of gene deletion DIVA(differentiating infected from vaccinated animals) vaccines [6–9], gene deletion mutants of the emerging PRV may be promising vaccine candidates for infection control and eradication [10].

Expectedly, PRV glycoprotein E (gE) gene is one target of deletion for differentiation purpose [11, 12]. For further attenuation, thymidine kinase(TK), glycoprotein I(gI) and/or glycoprotein G(gG) genes were also chosen as targets for deletion to generate gE&gI, TK&gE, TK&gE&gI or TK&gG deletion mutants [6, 13]. Several mutants of gE, gE&gI or gE&gI&TK deletion from the new variant have been generated and evaluated for safety and immunogenicity [11, 12, 14–19]. However, the safety and/or immunogenicity of these mutants is far from being satisfactory. TK is associated with virulence and reactivation from latent infection of PRV, and therefore the deletion of TK leads to attenuation of virulent PRV [20, 21]. As known, gE can form complexes with gI to obtain neuro-tropism and reactivation. It is proposed that gI will lose this function without gE, while immune stimulation by gI will be reserved. A TK&gE dual deletion mutant of the wild-type PRV TNL strain, which was isolated from a commercial pig farm in southern Taiwan in 1976, has been generated and proposed potential vaccine candidate with safety, efficacy and DIVA capability [22]. To our knowledge, no TK&gE deletion mutant from the new emerging Chinese variant has been reported so far.

Therefore, in this study, a TK&gE dual deletion mutant was constructed using a bacterial artificial chromosome clone of the emerging PRV AH02LA strain. Safety and immunogenicity of this mutant was evaluated in regard to its potency as a vaccine candidate for the control or eradication of the new emerging pseudorabies virus in Chinese pig herds.

## Methods

### Cells and viruses

Swine testicular (ST) cells (CVCC:CL27, from China Veterinary Culture Collection Center), and primary or secondary chicken embryo cells (CECs) made from 10 days SPF chicken embryo (from Beijing Merial Vital Laboratory Animal Technology Co., Ltd.) were cultured in Dulbecco’s Modified Eagle Medium(DMEM, Gibco) supplemented with 2~ 10% newborn calf serum(NCS, Gibco), 100 U/mL penicillin and 100 μg/mL streptomycin at 37 °C, 5% CO_2_ atmosphere. The PRV AH02LA strain was isolated and identified in our lab(CGMCC No. 10891) [23]. The gE deletion mutant(LA-A^B^) from AH02LA strain was constructed in our lab as reported previously [23]. The PRV Bartha K61 strain was kindly provided by Professor Ping Jiang at the Nanjing Agricultural University, China. Virus cultures and stocks were prepared using CECs or ST cells and were frozen in aliquots at -70 °C after three round of freeze-thawing(− 70 °C and 37 °C). Virus titers were determined by TCID_50_ on ST cells following the Reed-Muench method. Viral Deoxyribonucleic acid(DNA) was extracted from infected cells by the sodium dodecyl sulfate(SDS)-proteinase K method. Transfection of plasmid, virus or bacterial artificial chromosome(BAC) DNA were performed using Lipofectamine® 3000 (Invitrogen) following manual of supplier.

### Bacterial manipulation

The BAC of PRV AH02LA strain(BAC^PRV-G^), constructed previously in our lab [23], was used for generation of TK&gE deletion mutant. Plasmid and BAC DNAs were prepared with commercial kits (QIAGEN). Restriction fragment length polymorphism(RFLP) analyses of PRV BAC or BAC mutants was conducted using the restriction endonucleases *Kpn* I (Takara) as described earlier [24]. Electroporation was performed to transform plasmid or BAC DNA as previously described [24].

### PCR and sequencing

**Fig. 1:**
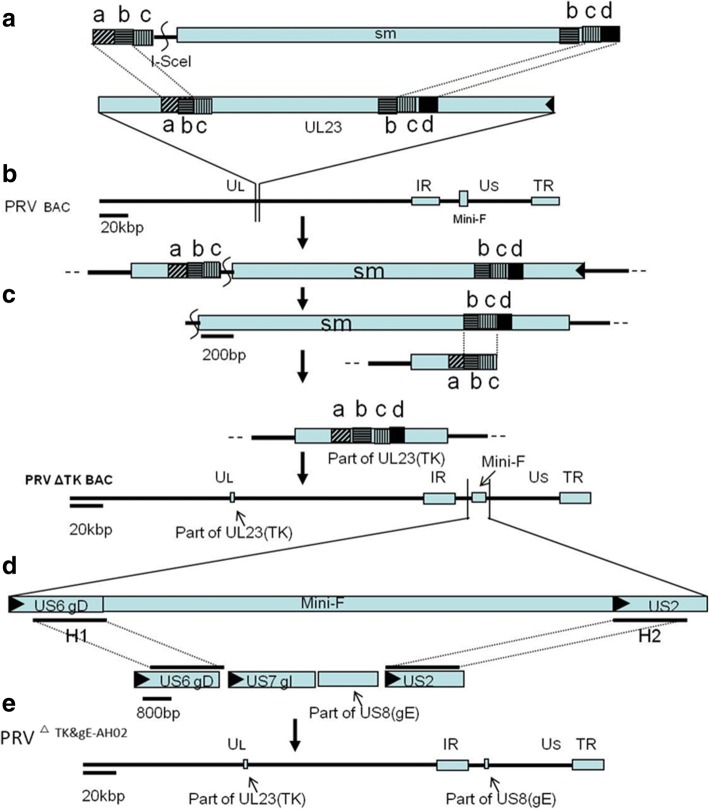
Construction of TK&gE dual deletion mutant virus (PRV^ΔTK&gE-AH02^ strain). **a** A fragment with selection mark(sm) was amplified to target part of UL23(TK) gene in the genome of PRV. **b** Homologous recombination was conducted through *En Passant* protocol to delete part of TK gene. **c** The sm of kanamycin resistance gene was removed in the 2nd Red recombination. **d** Another homologous recombination was performed to recover the whole gI gene and part of gE gene during virus rescuing. **e** Schematic presentation of the TK&gE dual deletion mutant was shown. Scales in bp or kbp are provided

To conduct *En Passant* recombination, a pair of primers (PRV ΔTK En pa F/R) for amplification of kanamycin resistance gene was designed with 60 bp homologous sequences (Fig. 1a)(Table 1) [25]. Primers of PRV ΔTK check F/R were used for check of correct insertion of kanamycin resistance gene and correct deletion of TK gene (Table 1). Primers(PRV BAC H1 F and PRV BAC H2 R) were designed from a reference sequence (GenBank:NC_006151.1) to amplify a DNA fragment including upstream and downstream homologous arms, the intact gI gene and part of gE gene(1299 bp to 1735 bp of gE ORF) (Fig. 1d) (Table 1) using isolated PRV LA-A^B^ strain DNA as template. The correct deletion of gE was verified with a pair of primers(PRV ∆gE check F/R)(Table 1). The primers used for sequencing gI gene were prepared as described previously(GeneScript, Nanjing China). [26]

**Table 1:** Primers for PCR or sequencing

Primer	Sequence(5′- 3′)	Positions
PRV ΔTK En pa F	CGGTATTTACGATGCGCAGACCCGGAAGCAGAACGGCAGCGCTCACGGCCCTGCGCAACGGGATGACGACGATAAGTAGGGATAAC	59,655
PRV ΔTK En pa R	GTTGACCAGCATGGCGTAGACGTTGCGCAGGGCCGTGAGCGCTGCCGTTCTGCTTCCGGGGGGTAATGCCAGTGTTACAACCA	60,081
PRV ΔTK check F	CGGATCTACCTCGACGGCGCCTA	59,524
PRV ΔTK check R	TTGTACGCGCCGAAGAGGGTGT	/
PRV BAC H1 F	GTACCCGTACACCGAGTCGT	121,170
PRV BAC H2 R	TTGTGGACCCGCGCGAACAT	126,648
PRV ∆gE check F	AGCCCCGGGAAGATAGCCAT	123,141
PRV ∆gE check R	ATCGCGGAACCAGACGTCGAAG	125,157
PRV gD part F	GGTGCGCGCACCTGCTGTACTTTA	121,385
PRV gD part R	AGATGTAGACGCACACGCCCACCAG	122,191

Positions indicate the position of the first base of 5′ terminal on the reference genome sequence NC_006151.1. “/” indicate the primer is not on the genome of PRV but the Kanamycin resistance mark gene

### Construction of PRV BAC deletion mutants

The PRV BAC TK deletion mutant was constructed from BAC^PRV-G^ following *En Passant* protocol as described previously (Fig. 1a, b and c) [27]. Briefly, after digestion with *Dpn* I, approximately 100 ng of purified DNA fragments amplified with primers of PRV ΔTK En pa F/R was electroporated into BAC^PRV-G^ at 1500 V/cm, a resistance and a capacitance of 25F(Precision Pulse, ECM630 BTX). Colonies with resistance to both chloramphenicol(34 μg/mL) and kanamycin(50 μg/mL) were checked through PCR with a pair of primers(PRV ΔTK check F/R) and through RFLP after digestion with *Kpn* I. One correct colony consistent with prediction from reference sequence of PRV ZJ01 strain (GenBank:KM061380.1) was used for the 2nd Red recombination to remove the kanamycin resistance gene (Fig. 1c). Colonies sensitive to kanamycin but resistant to chloramphenicol were double checked for the correct deletion of TK gene through PCR and RFLP as described above.

To generate the TK&gE deletion mutant of PRV AH02LA strain, another homologous recombination was performed to recover the gI gene and the undeleted sequences of gE (Fig. 1d). Briefly, PCR was carried out using primers PRV BAC H1 F and PRV BAC H2 F R (Table 1) and the PRV LA-A^B^ DNA as template. Primary CECs were co-transfected with approximately 1 μg of PRV BAC DNA and 100ngof DNA fragment from the PCR reaction using Lipofectamine® 3000 (Invitrogen™). Non-fluorescent virus plaques were purified after several round of picking and plating to obtain a homogeneous virus populations. Selected viruses were confirmed for correct deletion of TK gene and gE gene by PCR and sequencing using primers(PRV ΔTK check F/R and PRV ∆gE check F/R) (Table 1), and the recovery of gI gene were checked as described previously [23].

### Multistep growth kinetics

Multistep growth kinetics of parental AH02LA, gE deletion mutant (LA-A^B^ strain), and TK&gE dual deletion mutant (PRV^ΔTK&gE-AH02^) viruses were conducted following the methods as described previously with slight modifications [24]. Briefly, supernatant and cell-associated virus titers were determined before infection and at 6, 12, 24, 36, 48 and 72 h post infection (P.I.) on monolayers (1 × 10^6^ cells) of ST cells with a multiplicity of infection (MOI)of 0.01. Viruses from supernatant or released from cells by three freeze-thaw cycles were titrated on fresh ST cells. Three independent experiments were conducted and one-way ANOVA (SPSS software package 17.0, IBM SPSS, Chicargo, IL, USA) was employed for statistical analysis.

### Preparation of vaccine

PRV^ΔTK&gE-AH02^, PRV LA-A^B^ and Bartha K61 viruses were propagated on ST cells using a 5 l bioreactor, each virus stock was titrated by TCID_50_ following the Reed-Muench method on ST cells. All vaccines were confirmed to be free of bacteria and fungi following standard method as described in “People’s Republic of China Veterinary Pharmacopoeia, 2015 edition”, and were stored at -70 °C until use.

### Test of vaccine safety of PRV^ΔTK&gE-AH02^

Twenty one-day-old PRV gB antibody negative piglets (from Zhengzhuquan Pig Breeding Farm in Pukou district, Nanjing, China), free of porcine reproductive and respiratory syndrome virus (PRRSV), porcine parvovirus (PPV), porcine circovirus 2(PCV2) and classical swine fever virus (CSFV), were divided into 5 groups. In group A(N) and B(N) piglets were inoculated intranasally(I.N.) with PRV^ΔTK&gE-AH02^ and PRV LA-A^B^ respectively with a dose of 10^7.0^TCID_50_, while in group C(N) and D(N) intramuscularly (I.M.) respectively. Group E(N) was inoculated 2 mL PBS as placebo control (Table 2). Clinical signs were monitored daily for 14 days. Serum samples were collected for PRV gB and gE antibodies test at 14 days P.I..

**Table 2:** Groups division and results of antibodies, clinical signs in safety test

Groups			A(N)	B(N)	C(N)	D(N)	E(N)
Inoculation route			I.N.		I.M.		
PRV strain tested			PRV^ΔTK&gE-AH02^	LA-A^B^	PRV^ΔTK&gE-AH02^	LA-A^B^	
ELISA antibodies against PRV gB or gE	B.I.	gB+	0^a^/4^b^	0/4	0/4	0/4	0/4
	14d P.I.	gB+	3/3	/	4/4	1/1	0/4
		gE+	0/3	/	0/4	0/1	0/4
Morbidity			2/4	4/4	1/4	4/4	0/4
Mortality			1/4	4/4	0/4	3/4	0/4
Groups			A(P)	B(P)	C(P)	D(P)	E(P)
Inoculation route			I.N.		I.M.		
PRV strain tested			PRV^ΔTK&gE-AH02^	LA-A^B^	PRV^ΔTK&gE-AH02^	LA-A^B^	
ELISA antibodies against PRV gB or gE	B.I.	gB+	4/4	4/4	4/4	4/4	4/4
	14d P.I.	gB+	4/4	4/4	4/4	4/4	4/4
		gE+	0/4	0/4	0/4	0/4	0/4
Morbidity			0/4	0/4	0/4	0/4	0/4
Mortality			0/4	0/4	0/4	0/4	0/4

*I.N*. means intranasally, *I.M*. means intramuscularly, *gB+* means antibodies against PRV gB positive, *gE+* means antibodies against PRV gE positive, *B.I*. means before inoculation, *P.I.* means post inoculation. “^a^” indicates the number of piglets positive;“^b^” indicates the number of piglets in the group

Twenty one-day-old PRV gB antibody positive/gE antibody negative piglets(from Zhengzhuquan Pig Breeding Farm in Pukou district, Nanjing, China), also free of PRRSV, PPV, PCV2 and CSFV, were divided into 5 groups of A(P), B(P), C(P), D(P) and E(P). All piglets were treated and monitored exactly as those PRV gB antibody negative piglets (Table 2).

Safety of PRV mutants in mice (Balb/c mice, from Nanjing biomedical research institute of Nanjing University,Nanjing, China) was also tested. PRV^ΔTK&gE-AH02^, PRV LA-A^B^ and parental PRV AH02LA were separately used to inoculate mice subcutaneously with doses of 10^6.0^, 10^5.0^, 10^4.0^ and 10^3.0^ TCID_50_ respectively (Table 2). LD_50_ of each strain for mice were determined following Reed-Muench method. The euthanasia of survived mices mere performed usingy compressed CO2 gas.

### Test of vaccine efficacy of PRV^ΔTK&gE-AH02^

Twenty four 28~ 35-day-old piglets(from Zhengzhuquan Pig Breeding Farm in Pukou district, Nanjing, China) were randomly divided into 6 groups (A-F). All piglets were antibody negative for PRV gB and gE, and free of PRRSV, PPV, PCV2 and CSFV. Piglets in group A, B and C were vaccinated with PRV^ΔTK&gE-AH02^ strain with 1 × 10^3.0^, 1 × 10^4.0^ and 1 × 10^5.0^ TCID_50_ /dose respectively, group D piglets with live Bartha K61 of 1 × 10^5.0^ TCID_50_ /dose, and groups E and F piglets were dosed with PBS only(Table 3). All inoculations were given intramuscularly(I.M.) at 2 mL/pig. At 1 week post vaccination(P.V.), groups A, B, C, D and E were challenged intranasally (I.N.) with 2LD_50_ PRV AH02LA per piglet. Group F piglets were not challenged, serving as a negative control. Clinical signs, body temperature and virus shedding were monitored and recorded daily from vaccination to 14 days P.C.. Serum samples from all piglets were collected for PRV gB and gE antibodies test before vaccination, and at 7 days post vaccination (P.V.), and 14 days post challenge (P.C.). All survived piglets were euthanized by injection of pentobarbital. The presence of lung lesions for survived or died piglets was noted at the end of the test.

**Table 3:** Group division and results of antibodies, clinical signs and lung lesions in efficacy test

Groups			A	B	C	D	E	F
Virus tested			PRV^ΔTK&gE-AH02^			Bartha K61	/	/
Dosage (TCID_50_)			10^3^	10^4^	10^5^	10^5^	/	/
Inoculation route			Intramuscularly					
ELISA antibodies against PRV gB or gE	B.V.	gB+	0^a^/4^b^	0/4	0/4	0/4	0/4	0/4
		gE+	0/4	0/4	0/4	0/4	0/4	0/4
	7d P.V.	gB+	4/4	4/4	4/4	4/4	0/4	0/4
		gE+	0/4	0/4	0/4	0/4	0/4	0/4
	14 d P.C.	gB+	4/4	4/4	4/4	4/4	0/4	0/4
		gE+	4/4	4/4	4/4	4/4	1/1	0/4
Clinical signs P.V.			0/4	0/4	0/4	0/4	0/4	0/4
Fever frequency (≥40.5 °C) P.V.			0/4	0/4	0/4	0/4	0/4	0/4
Virus shedding P.V.			0/4	0/4	0/4	0/4	/	/
Fever frequency (≥40.5 °C) P.C.			0/4	0/4	0/4	4/4	4/4	0/4
Clinical signs P.C.	Morbidity		0/4	0/4	0/4	0/4	4/4	0/4
	Duration (days)		/	/	/	/	4~ 9	/
	Mortality		0/4	0/4	0/4	0/4	3/4	0/4
Virus shedding P.C.	Frequency		0/4	0/4	0/4	4/4	4/4	0/4
	Duration (days)		/	/	/	3~ 5	2~ 9	/
Lung lesions			0/4	0/4	0/4	2/4	4/4	0/4

*B.V.* means before vaccination, *P.V.* means post vaccination, *P.C.* means post challenge, *gB+* means antibodies against PRV gB positive, *gE+* means antibodies against PRV gE positive, “^a^” indicates the number of piglets positive; “^b^” indicates the number of piglets in the group

### Test for serological antibodies

ELISA tests were performed using PRV gE or gB antibody detection kit (IDEXX, Maine, USA) following the manufacturer’s instructions. In safety experiment, all 1-day-old piglets were tested for serum antibodies against PRV gB and gE, and then divided into PRV negative group or positive group accordingly. At 14 days post vaccination, serum samples of all piglets were collected for test of PRV gB and gE antibodies again. In efficacy experiment, serum samples of all piglets were collected before vaccination, at 7 days post vaccination (before challenge) and at 14 days P.C. for test of PRV gB and gE antibodies.

### Detection of virus shedding

Nasal swab samples were collected daily from all piglets in efficacy experiment from inoculation of vaccines to 14 days P.C.. Treatment and titration of nasal swab samples were performed following the method as described earlier. Viral DNA isolated from titration cells with typical cytopathogenic effects (CPE) was used as template to amplify a specific fragment of 813 bp in gD gene with a pair of primers(PRV gD part F/R) (Table 1).

Experiments involving virulent PRV were conducted under Biosafety Level 2+ containment. All animal tests were approved by the Experimental Animal Committee of the Jiangsu Academy of Agriculture Sciences and were conducted in accordance with the “Guidelines for Experimental Animals” of the Ministry of Science and Technology (Beijing, China). All animals were fed with complete formula feed and free access to drinking water.

## Results

### Generation of mutant PRV BAC with TK deletion

**Fig. 2:**
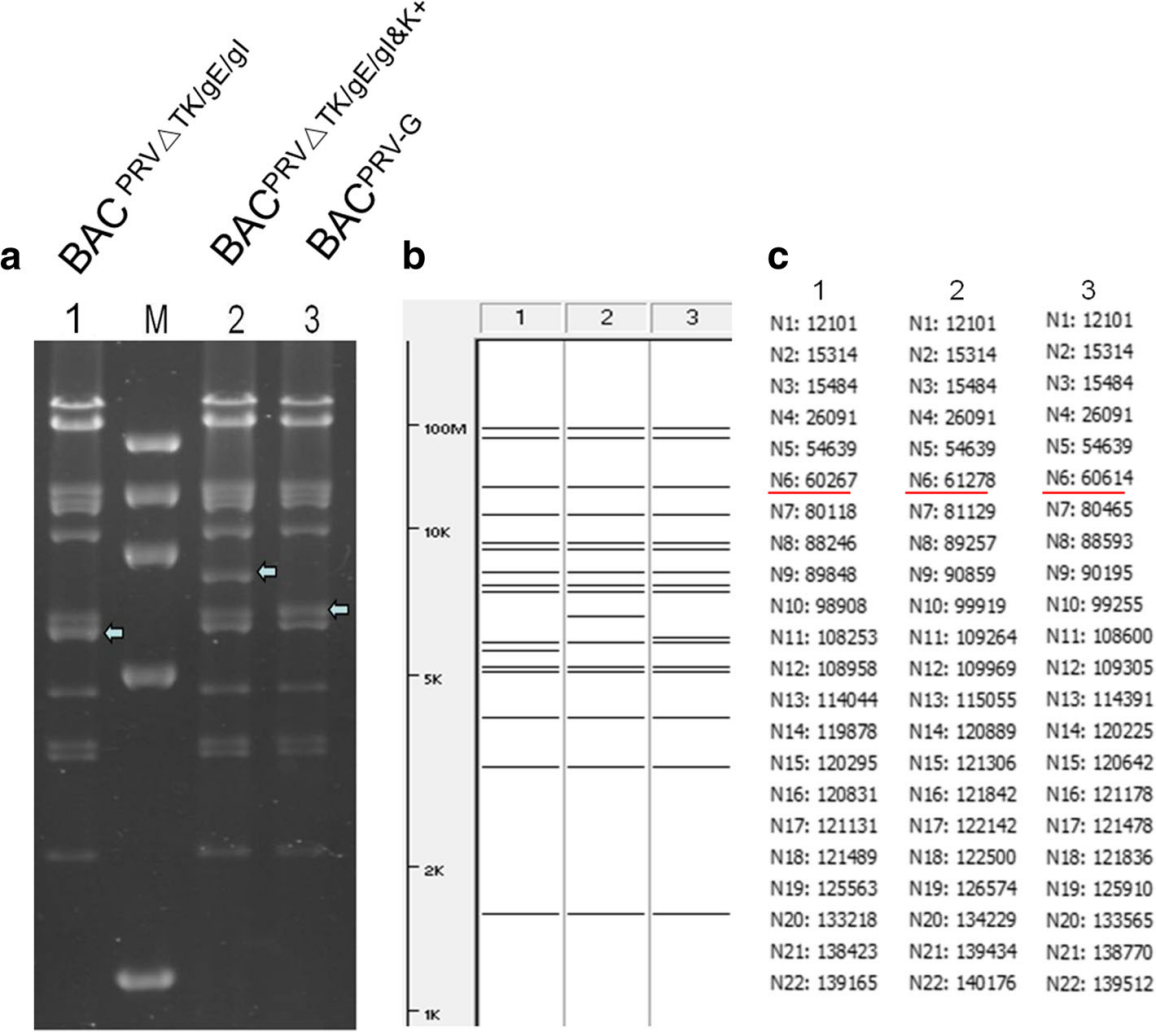
Restriction fragment length polymorphism (RFLP) of BAC^PRV-G^ and its TK deletion mutants. **a** RFLP pattern of BAC ^PRV△TK/gE/gI^, BAC^PRV△TK/gE/gI&K+^ and BAC^PRV-G^. Lane 1, 2 and 3 are BAC ^PRV△TK/gE/gI^, BAC^PRV△TK/gE/gI&K+^ and BAC^PRV-G^ respectively after digestion with *Kpn* I. The arrow (lane 2) shows an additional band of 6639 bp and a 5975 bp band missed when compared to lane 3. The arrow(lane 1) showed an additional band of 5628 bp and a missing band of 6639 bp when compared to lane 2. M is 1Kb DNA marker. **b** Predicted RFLP pattern with PRV ZJ01 strain (GenBank:KM061380.1) as a reference. Lane 1, 2 and 3 are prediction of BAC ^PRV△TK/gE/gI^, BAC^PRV△TK/gE/gI&K+^ and BAC^PRV-G^ respectively digested with *Kpn* I. **c** *Kpn* I sites were cited in lane 1, 2 and 3 for BAC ^PRV△TK/gE/gI^, BAC^PRV△TK/gE/gI&K+^ and BAC^PRV-G^ respectively. Sites underlined with red lines indicate the changed position of *Kpn* I restriction site leading to the bands changing accordingly

Based on BAC ^PRV-G^, we targeted a 347 bp fragment in TK gene. With a first Red recombination, 120 ng of gel-purified DNA of PCR product was electroporated into BAC^PRV-G^. The PCR product was amplified with a pair of primers(PRV ΔTK check F/R) that specified the homologous sequences to allow recombination. As a result the sequences from position 184 to 530 of TK gene was replaced with a kanamycin resistance cassette. Several colonies with resistance to chloramphenicol and kanamycin were obtained and verified through PCR with a pair of primers(PRV ΔTK check F/R)(data not shown) and through RFLP after digestion with *Kpn* I(Fig. 2), which showed a band of 5975 bp missed and an additional band of 6639 bp present after the first recombination. One of the verified clones, named BAC^PRV△TK/gE/gI&K+^, was selected to perform the 2nd Red recombination for removal of the kanamycin resistance cassette. Colonies with resistance to chloramphenicol, but sensitive to kanamycin were selected and one of them, named BAC ^PRV△TK/gE/gI^, was successfully confirmed through PCR with a pair of primers(PRV ΔTK check F/R)(data not shown) and through RFLP after digestion with *Kpn* I(Fig. 2), which showed a missing band of 6639 bp and instead an additional 5628 bp band present. Finally, the sequencing of the resulting PCR products after removal of kanamycin resistance cassette confirmed the successful deletion of a fragment from position 184 to 530 of TK gene in the genome of BAC^PRV-G^.

### Reconstitution of a TK&gE dual deletion PRV from cloned DNA

**Fig. 3:**
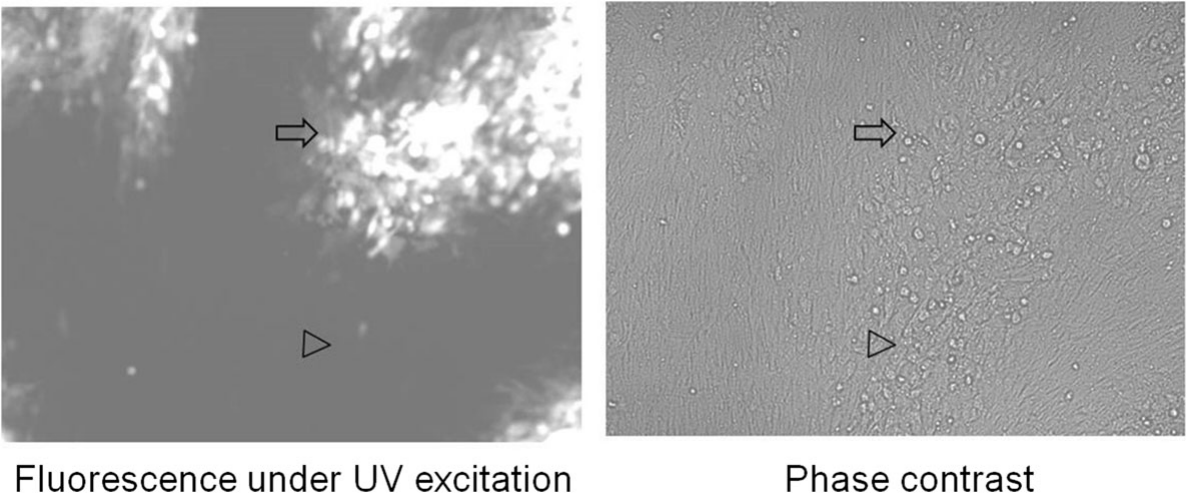
Plaques of TK&gE dual deletion mutant virus. Plaques are shown under UV excitation(left) or phase control(right). Arrow shows the plaque of rescued virus from BAC ^PRV△TK/gE/gI^. Arrowhead shows the plaque of TK&gE dual deletion mutant virus (PRV^ΔTK&gE-AH02^) after replacement of mini-F sequences through another homologous recombination. Individual panels present views of 600 × 600 μm

After co-transfection of BAC ^PRV△TK/gE/gI^ DNA with a DNA fragment that was amplified with primers PRV BAC H1 F and PRV BAC H2 F R (Table 1) using PRV LA-A^B^ DNA as template, non-fluorescing plaques were observed under UV illumination at 488 nm (Fig. 3). The recombinant virus was purified after several round of picking and plating, and designated PRV^ΔTK&gE-AH02^. The deletion of TK and gE and the recovery of gI were verified by PCR using primers of PRV ΔTK check F/R and PRV ∆gE check F/R respectively (Table 1). Sequencing with appropriate primers revealed the correct deletion of the 347 bp fragment in TK gene and the deletion of the 1286 bp fragment(position 13 to 1298) in gE gene and successful recovery of gI gene.

### Growth kinetics of the PRV^ΔTK&gE-AH02^ virus

**Fig. 4:**
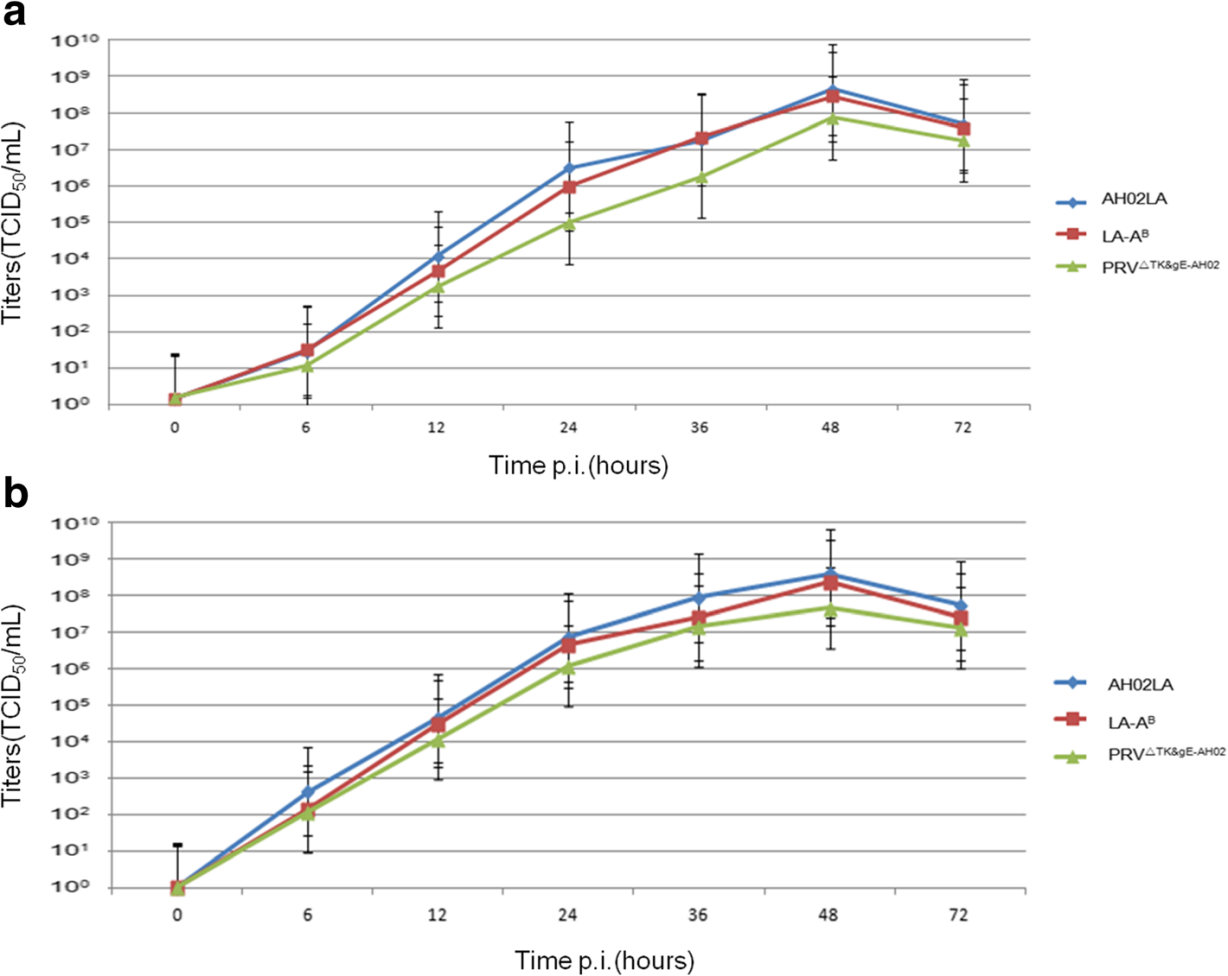
Multi-step growth kinetics of AH02LA, LA-A^B^ and PRV^ΔTK&gE-AH02^ on ST cells Titers of infected-cell supernatants (**a**) and cell-associated virus(**b**) of AH02LA, LA-A^B^ and PRV^ΔTK&gE-AH02^ were tested at 0, 6, 12, 23, 36, 48 and 72 h post infection with an MOI of 0.01. Shown are means of titers in three independent experiments. Standard deviation are shown with the error bar

Multistep growth kinetics for the PRV AH02LA, LA-A^B^, and PRV^ΔTK&gE-AH02^ viruses were determined on ST cells and the differences of titers at 48 h P.I. were statistically analyzed. For supernatant virus, peak titers for AH02LA, LA-A^B^, and PRV^ΔTK&gE-AH02^ were 10^8.81^, 10^8.71^, and 10^8.19^ TCID_50_ /mL respectively. At 48 hpi the titers of PRV^ΔTK&gE-AH02^ were not significantly different from LA-A^B^ (*p* = 0.212), while significantly different from AH02LA(*P* = 0.042). For cell- associated virus, peak titers of AH02LA, LA-A^B^, and PRV^ΔTK&gE-AH02^ were 10^8.71^, 10^8.49^, and 10^7.81^ TCID_50_ /mL respectively. At 48 hpi the titers of PRV^ΔTK&gE-AH02^ were significantly different from LA-A^B^ (*p* = 0.016), and AH02LA(*P* = 0.001). The results indicate that even though the mutant virus PRV^ΔTK&gE-AH02^ can not propagate on ST cells as efficiently as LA-A^B^ or AH02LA strain after deletion of TK gene, it reaches titers of more than 10^8.00^TCID_50_/mL (Fig. 4).

### Safety of the PRV^ΔTK&gE-AH02^ vaccine

**Fig. 5:**
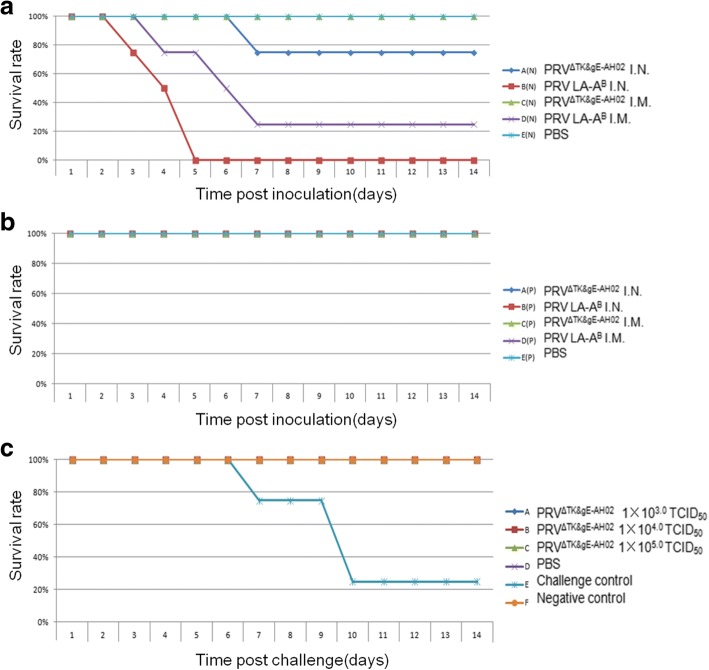
Safety and efficiency of TK&gE dual deletion mutant virus for piglets **a** Safety for 1-day-old PRV gB antibody negative piglets were tested. **b** Safety for 1-day-old PRV gB antibody positive piglets were tested. **c** A total of 24 28~ 35-day-old PRV gB antibody negative piglets were randomly divided into six groups of A-F. One week post vaccination, groups A, B, C, D and E were challenged intranasally with 2LD_50_ PRV AH02LA per piglet. Group F piglets were not challenged. The survival rates of these groups over the 14 days post challenge are shown. I.N.: intranasally; I.M.: intramuscularly

For 1-day-old PRV antibody negative piglets, 2 of 4 piglets in group A(N) showed typical clinical signs of PR from 3 days after inoculation intranasally with PRV^ΔTK&gE-AH02^, and one of the two ill piglets died at 7 days P.I. All the 4 piglets in group B(N) showed typical clinical signs of PR from 2 to 3 days after intranasal vaccination with PRV LA-A^B^ and all died at 3 to 5 days P.I.. In groups C(N) and D(N), 1 out of 4 and 4 out of 4 showed PR signs from 3 to 4 days post vaccination intramuscularly with PRV PRV^ΔTK&gE-AH02^ or LA-A^B^. No animal died in group C(N),while 3 out of 4 died in group D(N) at 4 to 7 days P.I.. No PRV gE antibodies was detected in all survived piglets. All animals in placebo control group(E(N)) were healthy during the whole test(Table 2)(Fig. 5).

For 1-day-old PRV antibody positive piglets, neither PRV^ΔTK&gE-AH02^ (group A(P) and C(P)) nor LA-A^B^ (group B(P) and D(P)) showed any virulence post inoculation either intranasally or intramuscularly(Fig. 5). No PRV gE antibody was detected in all animals at 14d P.I. (Table 2).

The virulence to mice was reduced after deletion of gE gene and attenuated further after TK&gE dual deletion. LD_50_ to mice of AH02LA was 10^3.66^TCID_50_, and went up to 10^5.29^TCID_50_ for LA-A^B^ strain, while PRV^ΔTK&gE-AH02^ did show virulence to mice at a dose of 10^6.00^TCID_50_.

### Immunogenicity of the PRV^ΔTK&gE-AH02^ vaccine

In group E (challenge control), all piglets showed clinical signs of pseudorabies infection including sneezing, coughing, nasal discharge, respiratory distress, lack of coordination, circling and paralysis from 2 days to 11 days P.C.. One piglet died at 8 days and two at 10 days P.C. (Table 3) (Fig. 5). Body temperatures of all these piglets reached over 40.5 °C and lasted for 4~ 10 days (data not shown). All piglets shed virus from 2 or 3 days P.C. and lasting for 5~ 7 days till death or 6~ 10 days for the surviving piglets. PCR using primers((PRV gD part F/R)(Table 1)) with the DNA of isolated virus as template amplified a specific fragment of 813 bp as suspected (data not shown). All piglets showed severe lung lesions from hemorrhaging and congestion (Table 3). All animals in group F(placebo control) were healthy during the whole test(Table 3).

At 7 days post vaccination(P.V.), gB antibodies in all piglets vaccinated with the PRV^ΔTK&gE-AH02^ strain or Bartha K61 strain were detected positive. All piglets in vaccination groups A, B, C and D or survived piglets in group E were positive for gE antibodies at 14 days P.C. (Table 3). All animals in group F(placebo control) were gE and gB negative during the whole test(Table 3).

All piglets in the vaccinated groups (A, B, C and D) were protected against lethal challenge(Fig. 5). No fever, virus shedding or other clinical symptoms were observed in group A, B and C vaccinated with PRV^ΔTK&gE-AH02^ strain. While all four piglets in group D vaccinated with Bartha K61 strain showed fevers of ≥40.5 °C and virus shedding was detected for 3~ 5 days, even though no other clinical signs were observed in this group(Table 3). Special fragments of 813 bp were amplified through PCR with DNA of isolated virus as template and with primers(PRV gD part F/R). No lung lesions were observed in piglets of groups A, B or C at 14 days P.C., while 2 out of 4 piglets in group D and all group E piglets presented lesions from hemorrhage and/or congestion after death or euthanization at 14 days P.C.(Table 3).

## Discussion

Owing to the implementation of DIVA based vaccine [9], PRV eradication has been achieved with remarkable success [6]. Some developed countries including USA and several European nations have declared PRV free in domestic swine populations. In China, PRV was well controlled through intensive vaccination of traditional vaccines including Bartha K61 until late 2011, when a novel PRV variant with enhanced virulence emerged in several pig herds [2, 3]. AH02LA strain was isolated from the brain of a dead 1-day-old piglet in a pig farm during a PR outbreak in Anhui province of China in 2012 [23]. Sequence analysis reveals that AH02LA strain belongs to the same clade as the other new PRV variants isolated after 2011 in China [23, 28], and has significant mutations in gB, gD and gC genes when compared to the most popular vaccine strain Bartha K61 strain. Specially, gB (GenBank: KR605319) of AH02LA contains a 4 amino acid(aa) deletion at positions 75 to 78, a 4 aa insertion at positions 121 to 124, and 33 non-synonymous substitutions. The gC gene (GenBank: KR605320) of AH02LA contains a 2 nucleotide insertion at position 176 and 177, a 7 nucleotides insertions at position 184 to 190 and a 12 nucleotide insertion at position 196 to 207, leading to a 7 aa insertion, several aa changes, and 35 non-synonymous substitutions. The gD (GenBank:KR605321) of AH02LA contains a 2 aa insertion at positions 275 and 276 and 9 non-synonymous substitutions. gD, gB and gC are the major neutralizing stimulation antigens of PRV, the reduced protection efficiency of Bartha K61 vaccines against AH02LA strain might be closely associated with the mutations in these three genes. Theoretically, gene deletion mutants from homologous virulent virus could provide better protection.

To avoid the potential threat of the latent infection of PRV to porcine herds, the eradication strategy has been a direct selection of many countries or pig farms. DIVA vaccines have played an important role for control of disease and efficient reduction of virus shedding post infection with virulent strain. For differentiation purpose, gE deletion is the normal choice and the absence of gE can reduce the virulence of virus without affecting its immunogenicity and growth ability [29, 30]. However, absence of gE along can not be used as a live vaccine without further attenuation by an additional deletion of TK gene. In this study, a live vaccine candidate of TK&gE dual deletion strain(PRV^ΔTK&gE-AH02^ strain) from the emerging PRV AH02LA strain was constructed successfully. Safety and efficacy tests demonstrated significant attenuation of the PRV^ΔTK&gE-AH02^ strain virus and complete protection at 7 only days post vaccination. The PRV^ΔTK&gE-AH02^ strain had stopped virus shedding against challenge by virulent AH02LA strain, suggesting that it is superior to the Bartha K61 strain for the eradication of virulent virus. It is worth noting that the PRV^ΔTK&gE-AH02^ strain exhibited impaired replication in ST cells due to the deletion of TK and reached significantly reduced titers when compared to AH02LA or LA-A^B^ strain. Nevertheless, the peak titer of PRV^ΔTK&gE-AH02^ strain still reached 10^8.0^ TCID_50_ /mL which is sufficient for the production of a live vaccine.

In practice, 1-day-old piglets are inoculated intranasally(I.N.) with live vaccine in porcine farms that suffered from PRV emerging variants infection or under the threat of the infection. The safety test of PRV^ΔTK&gE-AH02^ strain for 1-day-old piglets demonstrated that both PRV^ΔTK&gE-AH02^ strain and LA-A^B^ strain are safe for PRV antibody positive piglets. However, although PRV^ΔTK&gE-AH02^ strain showed less virulence than LA-A^B^ in piglets without PRV antibody, it caused lethal infection after inoculation I.N. or I.M. These results indicate that further attenuation might be necessary to improve the safety of PRV^ΔTK&gE-AH02^. However, since farm pig herds are PRV antibody positive due to intensive vaccination of PRV vaccine and newborn piglets are surely PRV antibody positive after intake of sow’s colostrum, indicating that the PRV^ΔTK&gE-AH02^ strain should be safe for 1-day-old inoculation in farm pig herds.

Bacterial artificial chromosome(BAC) of PRV is a useful tool for the study of the virus [31–33]. After the construction of the first PRV BAC of Becker strain, a few of other PRV genomes have been maintained in BACs as infectious clones [14, 34, 35]. The infectious clone of the PRV AH02LA strain(BAC^PRV-G^) was previously constructed in our lab and was used in this study to generate the TK deletion BAC mutant by *En Passant* protocol. Additionally, this BAC can be used for the mechanistic analysis of the high virulence of this variant and the construction of other viral vectored vaccines.

## Conclusion

A TK&gE dual deletion mutant(PRV^ΔTK&gE-AH02^ strain) was generated successfully based on the BAC of the virulent PRV AH02LA strain. The PRV^ΔTK&gE-AH02^ strain was highly attenuated for 1-day-old piglets or mice. Live vaccine made of PRV^ΔTK&gE-AH02^ strain is safe for 4~ 5 week-old PRV antibody negative piglets and can provide complete protection against lethal challenge with the emerging PRV virulent strain AH02LA at only 7 days post vaccination. Most importantly, PRV^ΔTK&gE-AH02^ can stop virus shedding post lethal challenge and thus might be a promising vaccine candidate for the eradication of emerging virulent mutants of PRV in China.

## Abbreviations

aa: Amino acid
BAC: Bacterial artificial chromosome
CEC: Chicken embryo cell
CPE: Cytopathogenic effects
CSFV: Classical swine fever virus
DIVA: Differentiating infected from vaccinated animals
DNA: Deoxyribonucleic acid
ELISA: Enzyme-linked immunosorbent assay
gB: Glycoprotein B
gC: Glycoprotein C
gD: Glycoprotein D
gE: Glycoprotein E
gG: Glycoprotein G
gI: Glycoprotein I
I.M.: Intramuscularly
I.N.: Intranasally
LD_50_: 50% lethal dose
MOI: Multiplicity of infection
P.C.: Post challenge
P.I.: Post infection
P.V.: Post vaccination
PCR: Polymerase chain reaction
PCV2: Porcine circovirus 2
PPV: Porcine parvovirus
PRRSV: Porcine reproductive and respiratory syndrome virus
PRV: Pseudorabies virus
RFLP: Restriction fragment length polymorphism
TCID_50_: 50% tissue culture infectious dose
TK: Thymidine kinase
UV: Ultraviolet
